# Trained immunity: novel perspectives in diabetes and associated complications

**DOI:** 10.3389/fimmu.2025.1613602

**Published:** 2025-07-17

**Authors:** Yukun Liu, Yanqi Lei, Zhuojun Dai, Changfang Luo, Qiming Gong, Yanqun Li, Yong Xu, Wei Huang

**Affiliations:** ^1^ Department of Endocrinology and Metabolism, The Affiliated Hospital of Southwest Medical University, Luzhou, China; ^2^ Clinical Medical College of Southwest Medical University, Luzhou, China; ^3^ Metabolic Vascular Diseases Key Laboratory of Sichuan Province, Luzhou, Sichuan, China; ^4^ Sichuan Clinical Research Center for Nephropathy, Luzhou, Sichuan, China; ^5^ Sichuan Clinical Research Center for Diabetes and Metabolic Diseases, Luzhou, Sichuan, China

**Keywords:** trained immunity, diabetes, hyperglycemia, inflammation, epigenetics, metabolism

## Abstract

Recent studies have revealed that the innate immune system possesses the capacity to develop “trained immunity” via metabolic and epigenetic reprogramming, leading to non-specific memory responses distinct from the memory traditionally attributed exclusively to adaptive immunity. Hyperglycemia, acting as an initiating stimulus, drives myeloid progenitor cell proliferation and monocyte-derived macrophage expansion, which leads to a sustained pro-inflammatory phenotype that is closely associated with the pathogenesis of diabetes and its related complications. The paradigm of trained immunity provides a novel perspective on explaining the “metabolic memory” phenomenon in diabetes. Here, we summarize the research progress on trained immunity, diabetes, and related complications to explore novel insights into diabetes prevention and treatment.

## 1 Introduction

Diabetes encompasses a spectrum of metabolic disorders marked by hyperglycemia, now recognized as a global health crisis. Over the past three decades, its global prevalence has increased exponentially, escalating from approximately 200 million cases in 1990 to projections surpassing 500 million by 2025 (1). This dramatic escalation has positioned diabetes as a major public health challenge with significant socioeconomic implications.

The clinical impact of diabetes extends beyond hyperglycemia itself, manifesting as secondary systemic damage affecting cardiovascular (2), neurological (3) and renal systems (4). A particularly significant aspect is the “metabolic memory” phenomenon, wherein the adverse effects of early sustained hyperglycemia persist despite subsequent achievement of glycemic control, with disease-related risks remaining elevated (5). Current theoretical frameworks have not fully elucidated the molecular mechanisms underlying this persistence in diabetes-associated pathologies.

The paradigm of “trained immunity” has recently gained attention as a framework for examining “metabolic memory” in diabetes. While adaptive immunity has long been recognized for its immunological memory, trained immunity represents a distinct process in which innate immune cells develop a form of memory following exposure to specific stimuli such as pathogen-associated molecular patterns (PAMPs), damage-associated molecular patterns (DAMPs) (6, 7), or hyperglycemia (8). This process involves epigenetic and metabolic reprogramming, resulting in enhanced non-specific immune responses during subsequent encounters. The relationship between diabetes and trained immunity remains an emerging area of research with numerous unanswered questions. This review summarizes the research progress on trained immunity, diabetes, and related complications, while discussing potential therapeutic strategies that could provide a novel perspective for the future prevention and treatment of diabetic complications.

## 2 Overview of trained immunity

**Figure 1:**
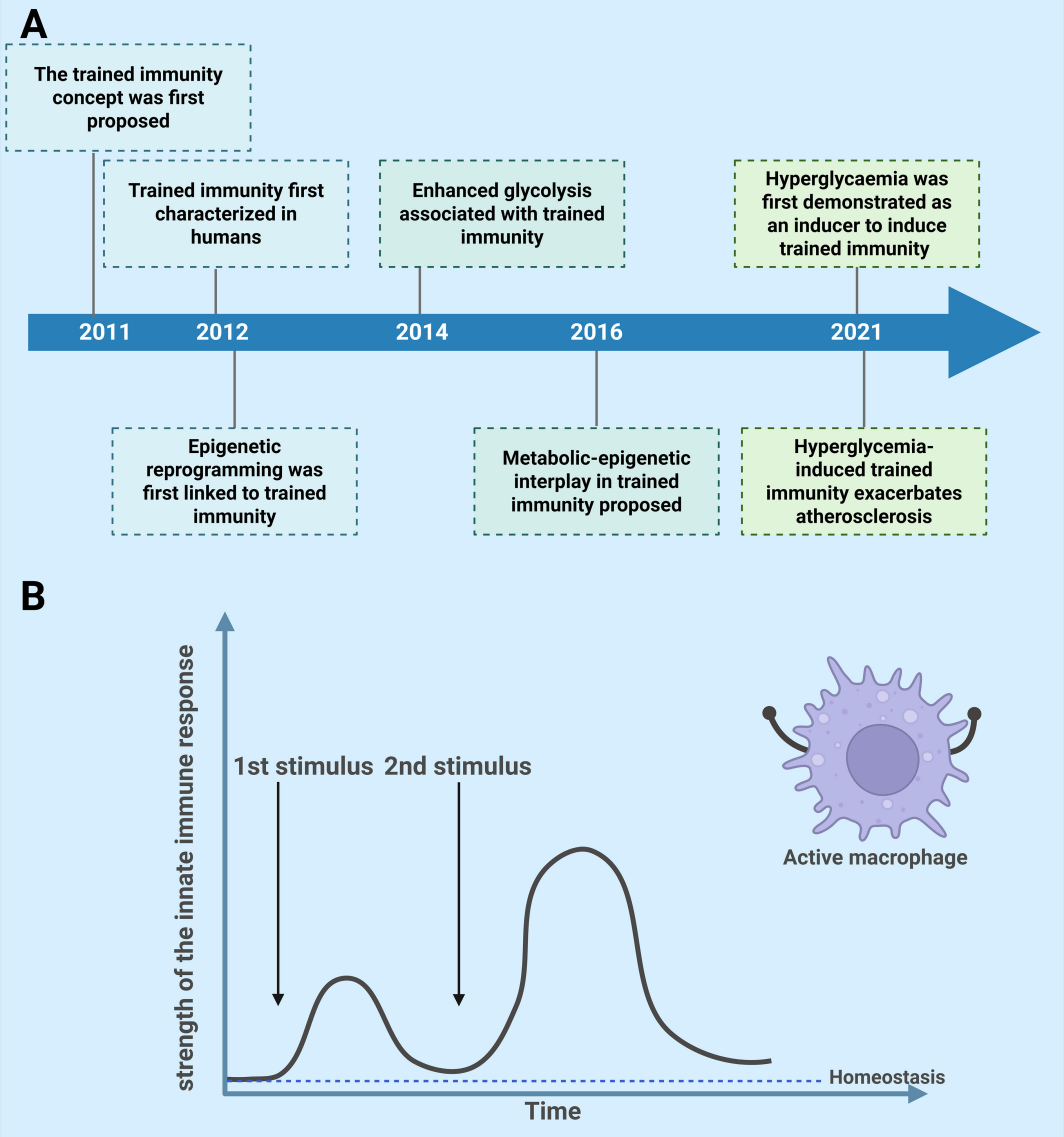
**(A)** A succinct historical overview of the development of trained immunity and hyperglycemia. **(B)** Hyperglycemia-mediated trained immunity. The first stimulus alters the functional state of macrophages, and their immune status fails to return to basal levels before the secondary stimulation or infection. High glucose priming of cells, followed by secondary stimulation with lipopolysaccharide or interferon-γ after a defined interval, amplifies immune responses, producing additive or synergistic effects compared to the original stimulus.

Substantial evidence has demonstrated the existence of immunological memory within the innate immune system, termed “trained immunity,” challenging the traditional view that memory is exclusive to adaptive immunity. The concept of trained immunity was initially introduced in 2011, defined as an augmented immune response of innate immune cells to subsequent challenges, attributed to the persistent effects of prior exposures (9). This phenomenon was first demonstrated in humans in 2012, revealing that Bacillus Calmette-Guérin (BCG) can functionally reprogram monocytes to exhibit a lasting enhanced phenotype (10) (**Figure 1A**).

Trained immunity constitutes the process through which innate immune cells acquire a form of immunological memory. When exposed to diverse stimuli, these cells develop distinct trained immunity phenotypes. For instance, treatment with the fungal ligand β-glucan confers protection against subsequent infections with *Staphylococcus aureus* (11, 12) while the peptidoglycan component muramyl dipeptide provides protection against *Streptococcus pneumoniae* and *Toxoplasma gondii* infections (13). Peripheral injection of lipopolysaccharide (LPS) induces trained immunity in microglia, which subsequently exacerbates ischemic brain damage 1 month after LPS challenge (14). Epidemiological evidence shows that live vaccines—including BCG, measles, smallpox, and oral polio vaccines—provide beneficial non-specific protection against infections beyond their target diseases (15–22), likely through trained immunity mechanisms.

Beyond pathogens, metabolic factors such as hyperglycemia(**Figure 1B**), Western-style diet (23), and endogenous molecules—including oxidized low-density lipoprotein (Ox-LDL) particles, lipoprotein(a), vimentin, and high mobility group box 1 (HMGB1)—can also induce trained immunity (24–26). However, these stimulating factors often trigger excessive immune responses, resulting in persistent inflammatory effects. Therefore, although trained immunity can confer certain benefits to the host organism, it may also exert detrimental effects in specific contexts (27).

## 3 The role and mechanism of hyperglycemia-induced trained immunity

**Figure 2:**
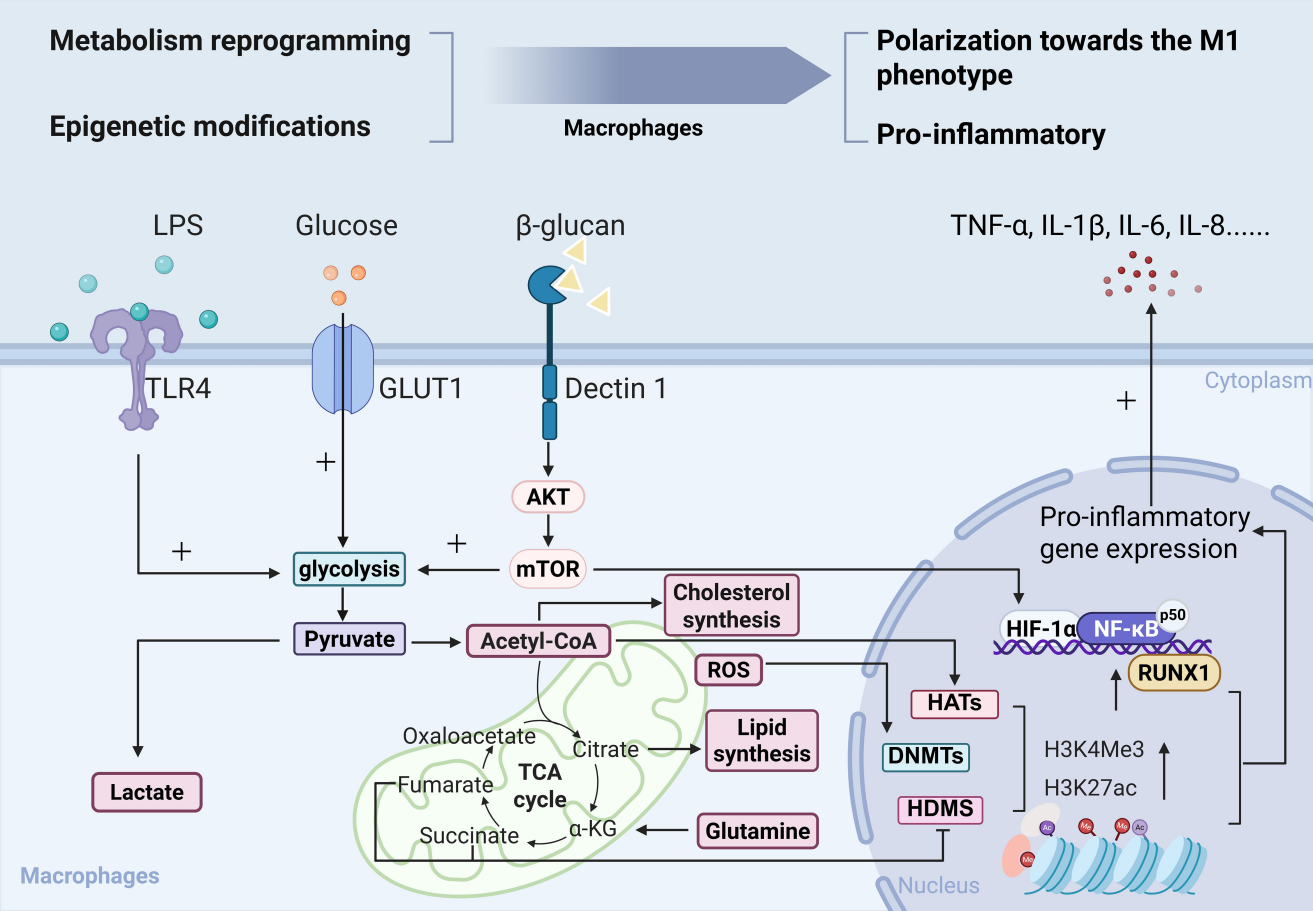
Macrophages undergo coordinated metabolic and epigenetic reprogramming. In macrophages, pro-inflammatory stimuli such as high glucose levels or lipopolysaccharide (LPS) enhance aerobic glycolysis and remodel the tricarboxylic acid (TCA) cycle, leading to altered levels of intermediate metabolites, such as increased reactive oxygen species (ROS), succinate, and acetyl-CoA. Enhanced glutaminolysis.These metabolic alterations directly modulate the activity of epigenetic-related enzymes, thereby influencing cellular function. β-glucan activates the AKT/mTOR/HIF-1α pathway through dectin-1 signaling, promoting aerobic glycolysis and subsequently mediating trained immunity. Additionally, RUNX1 binds to the NF-κB subunit p50, acting as a transcriptional co-activator to synergistically enhance TLR-4-induced production of IL-6 and IL-1β. Following metabolic reprogramming and epigenetic modifications, macrophages exhibit a greater tendency to polarize toward the M1 (pro-inflammatory) phenotype. GLUT1, glucose transporter 1; TLR4, toll-like receptor 4; AKT, protein kinase B; mTOR, mechanistic target of rapamycin; NF-κB, nuclear factor κB; p50, nuclear factor kappa-light-chain-enhancer of activated B cells subunit 1; DNMTs, DNA methyltransferases; HDMs, histone demethylases; HATs, histone acetyltransferases; α-KG, indicates α-ketoglutarate; RUNX1, Runt-related transcription factor 1; H3K4me3, histone H3 Lysine 4 trimethylation; HIF-1α, hypoxia inducible factor-1α; H3K27ac, histone H3 lysine 27 acetylation; TNF-α, tumor necrosis factor-α; IL-1β, interleukin-1β; IL-6, interleukin-6; IL-8, interleukin-8.

In diabetes, hyperglycemia activates trained immunity by expanding myeloid progenitors and releasing pro-inflammatory monocytes and neutrophils, thereby contributing to the progression of diabetic complications (8, 28, 29). The mechanisms underlying trained immunity primarily involve epigenetic and metabolic reprogramming (**Figure 2**), which are processes critical for establishing functional trained immunity in innate immune cells and their progenitors. For detailed insights into these mechanisms, several comprehensive reviews have been published (27, 30–32). Therefore, this review will not provide a detailed elaboration of these mechanisms.

### 3.1 Metabolic memory

Traditional mechanisms (33–37) of hyperglycemia-induced complications involve oxidative stress, polyol pathway activation, advanced glycation end products (AGEs) formation, protein kinase C pathway activation, and hexosamine pathway activation. However, these mechanisms inadequately explain the “metabolic memory” phenomenon. While previous research noted that AGEs accumulate in patients with long-term poor glycemic control and continuously exert pathological effects promoting vascular disease (33), this mechanism remains too generalized to explain the dynamic characteristics and individual variability of “metabolic memory”.

Clinical data from the Diabetes Control and Complications Trial (DCCT) demonstrated that hyperglycemic environments induce persistent epigenetic modifications in immune and tissue cells of type 1 diabetes (T1D) patients. Epigenetic markers such as H3K9Ac in monocytes significantly correlate with previous glycated haemoglobin (HbA_1c_) levels (38, 39), suggesting “metabolic memory” is closely linked to long-term epigenetic regulation (40, 41). However, traditional epigenetic explanations fail to clarify why “metabolic memory” persists for decades despite the relatively short lifespan and constant renewal of peripheral effector cells.

The trained immunity paradigm provides a novel framework for understanding the “metabolic memory” phenomenon. Hyperglycemia, as a trained immunity inducer, affects not only mature circulating immune cells but also crucially induces persistent metabolic and epigenetic reprogramming in hematopoietic stem cells (HSCs) and myeloid progenitor cells (8, 42, 43). This progenitor-level “memory” ensures that newly generated immune cells maintain pro-inflammatory phenotypes even after glycemic control is achieved, thereby explaining the continued progression of long-term complications. The trained immunity theory’s key advantage over other explanations lies in its focus on progenitor cell-level mechanisms and their intergenerational transmission.

Notably, glycemic variability—characterized by unstable fluctuations between peak and nadir blood glucose levels—is a common phenomenon in diabetes management (44). Clinical studies have shown an association between glycemic variability and the development and progression of diabetic complications (44–46). This pattern of intermittent hyperglycemic stimulation exhibits similarities to the initial stimulus and re-stimulation model characteristic of trained immunity. Glycemic variability likely triggers epigenetic and metabolic reprogramming in immune cells, inducing trained immunity that contributes to the establishment and maintenance of “metabolic memory”.

Taken together, diabetes impacts immune cell function via complex metabolic and epigenetic network remodeling, creating a regulatory network spanning metabolism, immunity, tissue homeostasis, and hematopoiesis. Diabetic patients, particularly those with type 2 diabetes (T2D), frequently present with comorbid conditions including hypertension (47), hyperlipidemia (48, 49), and obesity (50). Therefore, the trained immunity caused by other abnormal factors in diabetes and glycemic variability should also be given attention.

### 3.2 Epigenetic reprogramming

In trained immunity, non-permanent histone modifications are closely associated with gene activation. In the β-glucan-induced trained immunity model of macrophages, H3K4 monomethylation (H3K4me1) and trimethylation (H3K4me3) are significantly enriched in the enhancer regions of pro-inflammatory genes. This activation mechanism depends on upregulated expression of Set7 lysine methyltransferase. *In vitro* experiments confirm that Set7 inhibitors suppress pro-inflammatory memory effects induced by β-glucan (51, 52).

Hyperglycemia induces similar epigenetic remodeling in trained immunity. Mechanistic analysis reveals that high glucose promotes H3K4me3 deposition in pro-inflammatory gene promoter regions by upregulating the glycolytic pathway in monocytes and the mixed lineage leukemia (MLL) family of H3K4 methyltransferases. Clinical data further support that glycolysis-related genes and MLL methyltransferases are significantly upregulated in CD14^+^ monocytes of patients with T1D and THP-1 cells cultured under hyperglycemic conditions (42).

### 3.3 Metabolic reprogramming

Under steady-state conditions, immune cells display relatively low biosynthetic activity and predominantly rely on oxidative phosphorylation (OXPHOS) and fatty acid oxidation (FAO) for energy requirements. However, upon activation, innate immune cells undergo a substantial surge in energy demands. Consequently, aerobic glycolysis, glutaminolysis, cholesterol metabolism, and fatty acid synthesis become pivotal pathways to meet these elevated needs. This increased requirement for glucose and shift toward aerobic glycolysis resembles the “Warburg effect” observed in cancer cells (53, 54).

Elevated glucose levels drive macrophages toward glycolysis while reducing OXPHOS, a shift linked to protein kinase B (AKT) activation within the mechanistic target of rapamycin (mTOR) pathway (55). This metabolic reprogramming enables macrophages to secrete pro-inflammatory cytokines like tumor necrosis factor-α (TNF-α), interleukin-6 (IL-6), and interleukin-1β (IL-1β), perpetuating chronic inflammation.

Beyond macrophages, neutrophils also undergo metabolic reprogramming in diabetes, enhancing glycolysis via the pentose phosphate pathway and FAO. This leads to acetyl-coenzyme A accumulation, which, mediated by ATP-citrate lyase, promotes histone acetylation. Consequently, neutrophils form excessive neutrophil extracellular traps (NETs), thereby impairing wound healing in diabetic patients (28).

## 4 Trained immunity in diabetes and associated complications

**Figure 3:**
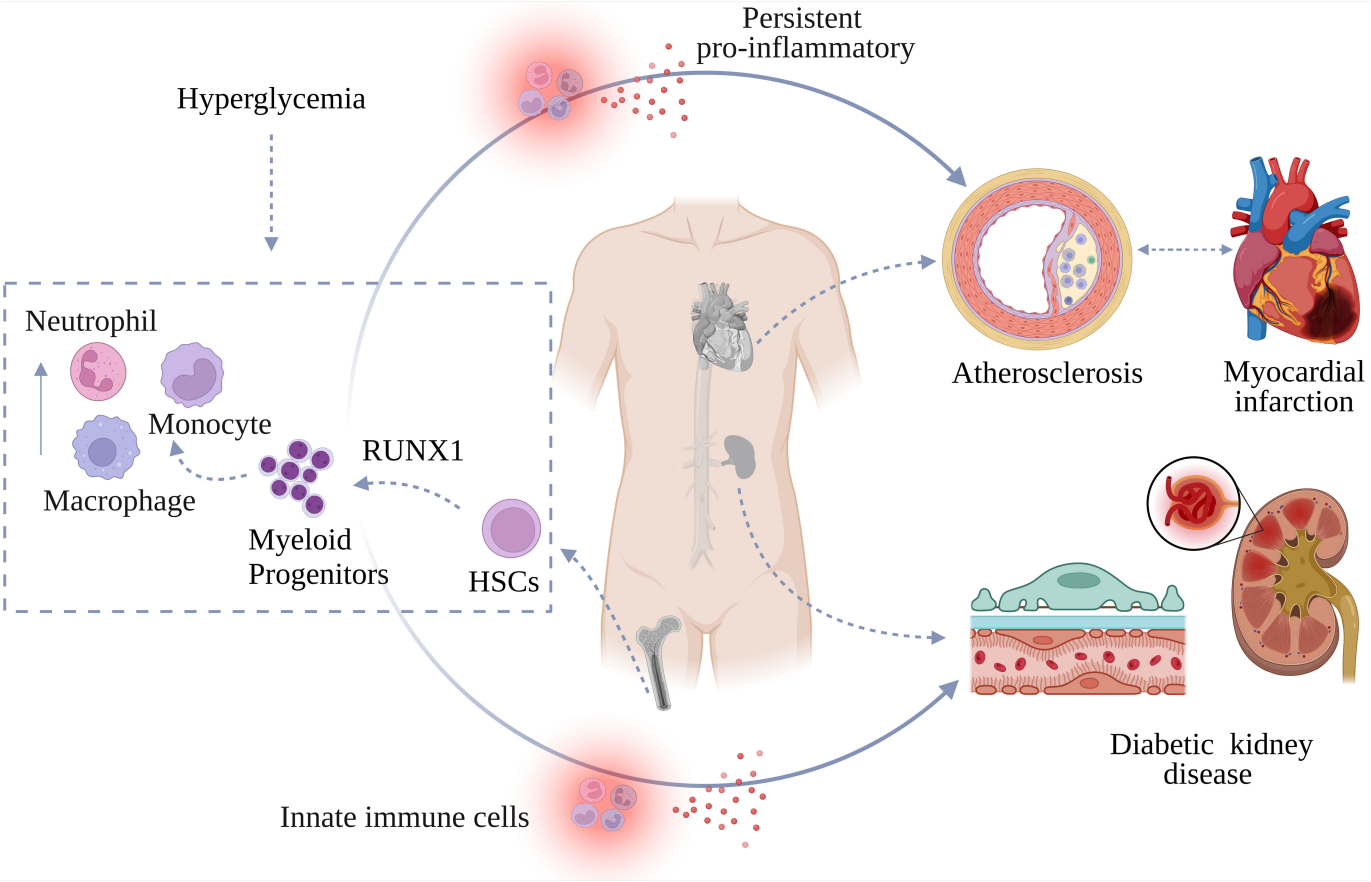
Trained immunity and vascular complications in diabetes. Hyperglycemia-induced trained immunity exacerbates vascular complications such as atherosclerosis, myocardial infarction (MI), and diabetic kidney disease by promoting hematopoietic stem cells (HSCs) differentiation and myeloid progenitor expansion differentiation, thereby increasing the release of innate immune cells, including pro-inflammatory monocytes and neutrophils. LPS, lipopolysaccharide; IFN-γ, interferon-γ; RUNX1, Runt-related transcription factor 1(It is mainly responsible for promoting the differentiation of HSCs, regulating the survival and differentiation of macrophages, and influencing the interaction between monocytes and endothelial cells).

Hyperglycemia induces persistent pro-inflammatory changes in HSCs that are transmitted to progeny cells, while simultaneously affecting mature cells in peripheral tissues (29). These dual processes lead to long-lasting pathological alterations in immune cell function and composition, accelerating vascular complications(**Figure 3**) even after glycemic control has been restored (29, 56).

### 4.1 Atherosclerosis

Atherosclerosis (AS), a chronic inflammatory vascular disease driven by genetic susceptibility, lifestyle factors, and systemic inflammation, contributes to significant global morbidity and mortality (57). Macrophages, central to atherosclerotic plaque pathogenesis, exhibit enhanced glycolysis, disrupted tricarboxylic acid cycle, and epigenetic alterations under hyperglycemic conditions (43, 58, 59). This cellular adaptation redirects their polarization toward a pro-inflammatory M1 phenotype while suppressing reparative M2 functions (60).

Animal studies show that even transient hyperglycemia accelerates AS through enhanced myelopoiesis (61). In 2021, a study conducted by the team led by Robin P. Choudhury provided robust evidence that hyperglycemia promotes trained immunity in HSCs and macrophages, significantly exacerbating AS (8). Central to this process is the transcription factor RUNX1, which orchestrates HSC differentiation, macrophage survival, and inflammatory programming (8).

Macrophages from hyperglycemic mice maintain enhanced cytokine production even after being cultured in normal glucose for 7 days. This inflammatory priming has significant *in vivo* consequences: bone marrow transplantation from hyperglycemic mice accelerates plaque formation in normoglycemic *LDL*-knockout mice (8). These plaques show H3K4me3 enrichment in macrophage-rich regions—a trained immunity marker absent in controls. Similar epigenetic and functional alterations are observed in leukocytes from T2D patients, confirming the clinical relevance of this phenomenon (8).

Notably, in hyperglycemic environments, all exposed tissue cells are affected, potentially inducing reprogramming in multiple cell types related to vascular health. Trained immunity characteristics have been documented in various immune and non-immune cells critical to AS, including dendritic cells (62), neutrophils (63), natural killer cells (64), vascular smooth muscle cells, (65) and endothelial cells (66). These findings suggest that trained immunity extends beyond innate immune cells, with hyperglycemia potentially inducing long-term vascular endothelial dysfunction through epigenetic reprogramming mechanisms across multiple cell types critical for vascular health.

### 4.2 Myocardial infarction

The pathogenesis of myocardial infarction (MI) is characterized by coronary artery obstruction, which subsequently leads to myocardial cell death due to ischemia and hypoxia. Diabetic patients face higher mortality and increased complications (reinfarction, heart failure, shock, arrhythmias), demonstrating hyperglycemia’s synergistic amplification of cardiac injury (67–72).

The post-MI inflammatory cascade is a tightly regulated yet complex process involving systemic and local immune activation. Bone marrow-derived immune cells are rapidly mobilized alongside resident cardiac immune responses, triggering the recruitment of circulating inflammatory cells critical for injury and repair. Neutrophils dominate the early phase (peaking at 24–48 hours post-MI), followed by macrophages, T/B cells, and dendritic cells, with macrophages playing dual roles in inflammation and tissue repair (53, 73).

In diabetic patients, hyperglycemia-induced trained immunity disrupts this balance, exacerbating post-MI inflammation. Experimental models demonstrate that Ly6C^Hi^ monocytes exhibit a pathological “second wave” of infiltration into ischemic myocardium, mirroring their delayed polarization to Ly6C^Lo^ phenotypes observed in diabetic wound healing (74). Some researchers speculate that this mechanism is likely to be related to the healing process of MI as well (75). Hyperglycemia further entrenches a pro-inflammatory macrophage phenotype, increasing their infiltration into ischemic tissue and suppressing reparative functions. The resulting inflammatory milieu not only delays healing but also heightens risks of adverse remodeling and heart failure.

Interestingly, a recent study has revealed that MI can act as a priming factor for monocytes to enhance trained immunity, thereby promoting the progression of AS (76). In patients with acute coronary syndrome (ACS), the expression of spleen tyrosine kinase (SYK) in monocytes may serve as a potential biomarker for predicting the risk of recurrent ischemic events. In this context, MI can be regarded as the “first hit,” while hyperlipidemia represents the “second hit.” Both conditions exert their effects through epigenetic modifications within the bone marrow and monocytes, jointly leading to increased SYK expression and maintenance of a persistent pro-inflammatory phenotype (76).

Based on these observations, a bidirectional interaction has been established between trained immunity and cardiovascular injury. Cardiovascular damage itself may initiate immune training via persistent inflammatory signaling. AS increases the risk of MI, while MI-induced immune priming subsequently exacerbates residual atherosclerotic lesions.

### 4.3 Diabetic kidney disease

The kidney serves as a key target organ for microvascular damage in diabetes. Diabetic kidney disease (DKD) pathogenesis is complex, arising from the interplay of multiple factors, including genetic predisposition, environmental influences, metabolic disorders, hemodynamic abnormalities, and immune responses. This pathological process is characterized by persistent hyperglycemia, immune complex deposition in the glomeruli, increased chemokine production, and macrophage recruitment (77, 78). These events trigger complex crosstalk between macrophages, non-myeloid cells, and adaptive immune cells. The inflammatory cascade is tightly linked to dysregulated macrophage function.

Notably, a growing body of evidence indicates that innate immune cells in the kidneys exhibit phenotypes consistent with trained immunity. Patients with chronic kidney disease (CKD) exhibit elevated CD14^++^CD16^+^ pro-inflammatory monocytes in bone marrow alongside heightened systemic levels of IL-6, IL-1β, and TNF-α, suggesting persistent innate immune activation (79). Monocytes stimulated by Ox-LDL and subsequently exposed to Toll-like receptor (TLR) 2 and TLR4 agonists demonstrate enhanced production of IL-6 and TNF-α, with upregulated H3K4me3 modification levels at inflammatory mediator gene promoters. This epigenetic modification is reversible by histone methyltransferase inhibition (24).

Environmental stressors further potentiate renal immune memory: high-salt diets exacerbate macrophage-mediated inflammation during secondary pathogen challenges, characterized by CD45^+^F4/80^+^ macrophage infiltration and cytokine surges that accelerate renal fibrosis (80). Uremic toxin accumulation in CKD, particularly indoxyl sulfate, activates trained immunity via aryl hydrocarbon receptor (AhR)-dependent arachidonic acid pathways, perpetuating inflammatory cascades (81). In experimental high-fat diet (HFD)+CKD models, synergistic lipid metabolism disturbances and caspase-11/LPS interactions upregulate 998 cytoplasmic genes linked to vascular inflammation via trained immunity mechanisms (82).

Although current research on hyperglycemia-induced trained immunity primarily focuses on the cardiovascular system, its specific manifestations and mechanisms in renal pathophysiology remain poorly understood. As a hallmark microvascular complication of diabetes, DKD pathogenesis likely involves trained immunity as a pivotal link connecting hyperglycemic memory to renal inflammatory injury.

### 4.4 Other diabetic complications involving trained immunity

In 1993, Loe first identified an elevated risk of periodontitis in diabetic patients, noting that it ranks as the sixth leading complication of diabetes (83). Subsequent epidemiological and intervention studies have demonstrated that individuals with diabetes are at a 3–4 times greater risk for developing periodontitis compared to those without diabetes (84–88).

Recent insights into trained immunity provide a novel framework for explaining the bidirectional relationship between diabetes and periodontitis. Systemic inflammation from periodontitis may activate trained immunity in peripheral immune cells and their precursors. Studies using 18F-fluorodeoxyglucose positron emission tomography-computed tomography (18F-FDG-PET/CT) imaging in patients with periodontitis support this hypothesis, showing an association between periodontitis and increased hematopoietic tissue activity (89, 90). Peripheral neutrophils in chronic periodontitis patients exhibit hyperresponsiveness with excessive reactive oxygen species (ROS) production (91) and increased pro-inflammatory cytokine release (92). Peripheral blood mononuclear cells from individuals with severe periodontitis also exhibit heightened IL-6 production (93). This cellular hyperreactivity persists even after successful periodontal treatment, aligning with characteristics of trained immunity.

Periodontitis and diabetes may reciprocally amplify inflammatory responses via trained immunity mechanisms. Both conditions induce sustained reprogramming in myeloid cells and their progenitors (94, 95). Specific bacterial products or inflammatory mediators can activate both peripheral myeloid cells and their bone marrow precursors, enhancing their responsiveness to subsequent challenges.

This bidirectional interaction creates a pathological feedback loop in which periodontal inflammation can exacerbate diabetes-associated immune responses, while hyperglycemia-primed cells exhibit heightened reactions to periodontal pathogens. This inflammatory interaction may contribute to the progression of both conditions. Current evidence indicates that trained immunity links oral and systemic inflammation, highlighting the need for integrated clinical management of these interconnected conditions.

## 5 Targeting trained immunity: emerging therapeutic prospects for diabetes and its complications

Trained immunity, originally established through BCG vaccination studies, now encompasses mechanisms mediated by both bone marrow progenitors and peripheral myeloid cells. While this enhanced adaptability improves antimicrobial and antitumor responses, its dysregulation can trigger pathological inflammation (27). This dual nature necessitates precise regulation of immune memory pathways.

For diseases where trained immunity deficiency promotes pathogenesis (certain cancers and infections), augmenting immune responses is the primary therapeutic strategy (96). Conversely, cardiovascular diseases and autoimmune disorders often exhibit excessive trained immunity-driven inflammation, thus requiring targeted anti-inflammatory approaches to restore immune homeostasis (96). We summarize current research directions in trained immunity-related therapeutics, including vaccines, nanomedicine, metabolic pathway modulation, and epigenetic interventions. Although direct research on trained immunity therapies for diabetes remains limited, these approaches may reveal potential targets for modulating diabetes-associated metabolic inflammation.

### 5.1 Vaccines

Vaccine-mediated trained immunity can induce enhanced innate immune responses against unrelated pathogens, providing non-specific protection, known as heterologous effects (97). Utilizing these heterologous effects, vaccines in the context of trained immunity are applied not only for infection prevention but also for regulating immune dysregulation diseases. Animal studies have demonstrated that BCG vaccination prevents candidiasis in severe combined immunodeficiency mice (98). Human research has confirmed that BCG-induced trained immunity provides non-specific protection against controlled human malaria (99) and experimental viral infections (100). For immunologically dysregulated tumors, BCG has been approved for intravesical administration in the treatment of non-muscle invasive bladder cancer (101). These studies highlight the therapeutic potential of vaccines in the field of trained immunity.

T1D is an autoimmune disease characterized by progressive destruction of pancreatic β cells (102), with pathological features including immune dysregulation and loss of self-tolerance. Current immunotherapeutic strategies for T1D primarily focus on targeting specific T and B cells to prevent islet β cell destruction. Treatment approaches such as anti-CD3 monoclonal antibody (Teplizumab) (103, 104) and anti-CD20 monoclonal antibody (Rituximab) (105) have shown certain efficacy. However, research on preventing T1D through targeted immune approaches remains relatively limited. The innate immune system, as the “first line of defense “ (106) and a key regulator of immune responses, has therapeutic potential through trained immunity. This approach, which targets innate immune cells to regulate immune tolerance or correct immune dysregulation, also deserves attention.

Epidemiological studies report that vaccination with the inactivated influenza vaccine Pandemrix^®^ reduces T1D risk in specific populations, suggesting that vaccine-induced trained immunity may participate in autoimmune regulation (107–110). An 8-year randomized study reported that double-dose BCG treatment could normalize HbA_1c_ in T1D patients after three years (111). Additionally, patients receiving BCG treatment exhibited a systemic metabolic shift from OXPHOS to aerobic glycolysis, consistent with trained immunity characteristics as confirmed in mouse experiments (111). BCG also restores insulin secretion and regulates immunity by inducing regulatory T cells and reducing autoreactive T cells (112).

Although these studies establish connections between vaccines and T1D through trained immunity, in-depth elucidation of the relevant immunomodulatory mechanisms remains insufficient, while research applications in T2D are considerably more limited. For T1D, future strategies could integrate adaptive immune-targeted monoclonal antibodies with innate immune interventions to achieve synergistic effects, enhancing preventive and therapeutic outcomes.

### 5.2 Nanomedicine

Nanomedicine is a rapidly evolving field that integrates nanotechnology, biomedicine, and pharmaceutical sciences (113). Nanoparticles, the fundamental components of nanomedicine, are biocompatible and biodegradable spherical systems that encapsulate conventional or biological drugs. They function as drug delivery vehicles that protect therapeutic agents from degradation at the administration site, facilitate targeted transport to specific tissues or organs, and enable controlled drug release in response to environmental stimuli at the target location (114).

The persistent effects of trained immunity originate from metabolic and epigenetic reprogramming of bone marrow progenitor cells, generating myeloid cells with enhanced responsiveness, termed “trained” myeloid cells (115, 116). This requires technologies capable of directly targeting myeloid progenitor cells (96). Given that nanomaterials inherently interact with phagocytic myeloid cells, nanomedicine provides an ideal platform for modulating trained immunity (117), enabling precise and efficient targeting of cells and inflammatory signaling pathways associated with trained immunity.

For example, nanoformulations loaded with mTOR inhibitors (mTORi-NB) can inhibit the production of pro-inflammatory cytokines in human monocytes stimulated with Ox-LDL (118). In experimental models, one-week treatment with mTORi-NB in *ApoE^-/-^* mice fed a Western diet for 12 weeks attenuated plaque inflammation (117). Such anti-inflammatory nanotherapeutic approaches may have potential applications in diabetes management.

Nanomedicine can modulate trained immunity at cellular, metabolic, and epigenetic levels through diverse material technologies, enabling precise immune modulation (117). However, translational applications in diabetes require further investigation of nanoparticle biocompatibility, cellular uptake, and drug release in diabetes-specific microenvironments.

### 5.3 Metabolic and epigenetic regulators

Metabolic and epigenetic reprogramming interact in trained immunity, with metabolic intermediates functioning as substrates, cofactors, or signaling molecules that regulate chromatin-modifying enzymes, establishing immunological memory. Given the role of metabolic alterations in driving the epigenetic foundations of trained immunity, targeting key metabolic enzymes to inhibit excessive trained immune responses represents a promising anti-inflammatory strategy.

Enhanced glycolysis, a critical metabolic signature of trained immunity, can be modulated by hexokinase inhibitors such as 2-deoxy-D-glucose (119) or mTOR pathway inhibitors including rapamycin and metformin (55). Glutamine catabolism is also upregulated during trained immunity. Succinate derived from glutaminolysis promotes pro-inflammatory histone modifications by inhibiting histone demethylase (HDM) activity, a process blocked by the glutaminase inhibitor BPTES(bis-2-(5-phenylacetamido-1,3,4-thiadiazol-2-yl)ethyl sulfide) (120). In addition, statins inhibit HMG-CoA reductase and attenuate β-glucan-induced trained immunity by depleting mevalonate pathway intermediates essential for epigenetic-modifying enzymes (121). Itaconate, produced via decarboxylation of cis-aconitate catalyzed by immunoresponsive gene 1, drives macrophage polarization toward an anti-inflammatory phenotype by inhibiting histone demethylases (122, 123).

Beyond metabolic targets, emerging pharmacological strategies focus on modulating key epigenetic modifiers in specific cellular or pathological contexts, including DNA methyltransferases (DNMTs), lysine methyltransferases (KMTs), and histone deacetylases (HDACs). For instance, DNMT inhibitors and HDAC inhibitors can reverse the silencing of pro-inflammatory genes, whereas activators of specific KMTs may regulate anti-inflammatory signaling pathways via modulation of histone methylation patterns (124–130).

Strategies targeting the metabolic-epigenetic axis offer multi-level intervention points for regulating trained immunity, but their clinical translation requires addressing drug specificity, tissue selectivity, and long-term safety considerations.

## 6 Conclusions and prospects

Hyperglycemia induces trained immunity in innate immune cells via epigenetic and metabolic reprogramming. Diabetic patients exhibit sustained functional alterations in monocytes and macrophages, thereby driving chronic inflammatory processes underlying complications (**Table 1**) such as AS and MI. The trained immunity paradigm provides a novel perspective on the “metabolic memory” phenomenon, offering a mechanistic framework that has significantly enhanced our understanding of diabetic immunopathology. Diabetes-related trained immunity demonstrates interconnectedness across various complications, forming a complex pathological network in which cardiovascular disease serves both as a target of trained immunity and as an activator of these pathways, thereby creating a self-reinforcing pathological cycle.

**Table 1:** Research summary of trained immunity with emphasis on diabetes-related studies.

Category	Model	Metabolic/Epigenetic Reprogramming Markers	Efficacy	Ref
*In Vivo*	*Ldlr^−/−^* mice transplanted with diabetic CD68-GFP BM and fed Western-style diet (12 weeks)	H3K4me3↑	Increased AS of the aortic root	(8)
	STZ-hyperglycemic C57BL/6 mice	Not Mentioned	TNF-α↑	(42)
	TIH *ApoE^−/−^* mouse model	GLUT-1↑Glycolysis↑	Myelogenesis↑Ly6-C^hi^ monocytes↑Neutrophils↑S100A8/A9-RAGE↑Accelerates AS	(61)
	(*ApoE* ^−/−^) C57BL/6J and BM chimeric mice underwent MI/IR followed by 12-week HFD	H4K20me↑	SYK↑KMT5A↑CNBP↑Monocyte pro-inflammatory in accelerated AS after MI	(76)
*In Vitro*	Human monocytes (under the Ox-LDL)	H3K4me3↑	TLR2/4-ERK/PI3K↑IL-6↑TNF-α↑Foam cell↑	(24)
	Murine BMDMs/HSCs (STZ-induced diabetic model) and human monocytes	Glycolysis↑H3K4me3↑H3K27ac↑	RUNX1↑IL-6↑IL-β↑Enhanced M1macrophage polarization	(8)
	BMDMs from diabetic mice	Glycolysis↑	TNF-α↑KC↑	(42)
	Human monocytes(under the hyperglycemia)	Glycolysis↑ H3K4me3↑	MLL gene family↑TNF-α↑ IL-6↑	(42)
	ESRD human monocytes (IS-induced stimulation)	Glycolysis↑ H3K4me3↑	AhR—ALOX5AP↑TNF-α↑IL-6↑	(81)

STZ, Streptozotocin; BM, bone marrow; H3K4me3, histone H3 Lysine 4 trimethylation; AS, atherosclerosis; TNF-α, tumor necrosis factor-α; TIH, transient intermittent hyperglycemia; GLUT1, glucose transporter 1; S100A8/A9, calprotectin, a heterodimeric Ca2^+^-binding protein mainly released by neutrophils; RAGE, receptor for advanced glycation end-products; BMDMs, bone marrow-derived macrophages; MI, myocardial infarction; IR, ischaemia–reperfusion; SYK, spleen tyrosine kinase; KMT5A, lysine methyltransferase 5A; CNBP, cellular nucleic acid-binding protein; HFD, high-fat diet; Ox-LDL, oxidized low-density lipoprotein; TLR2/4, toll-like receptor-2/4; ERK, extracellular signal-regulated kinase; PI3K, phosphatidylinositol 3-kinase; IL-6, interleukin-6; HSCs, haematopoietic stem cells; H3K27ac, histone H3 lysine 27 acetylation; RUNX1, runt-related transcription factor 1; IL-β, interleukin-β; KC, keratinocyte-derived chemokine; MLL, mixed lineage leukemia; ESRD, end-stage renal disease; IS, indoxyl sulfate; ESRD, end-stage renal disease; AhR, aryl hydrocarbon receptor; ALOX5AP, arachidonate 5-lipoxygenase (ALOX5) and ALOX5 activating protein; ↑, upregulation.

Given this pathological complexity, approaches targeting trained immunity in diabetes offer promising therapeutic prospects. Vaccines, nanomedicine, and metabolic-epigenetic modulators demonstrate certain therapeutic potential but require extensive diabetes-specific research to address clinical translation challenges. Importantly, these approaches can function synergistically, with nanomedicine serving as an integrative platform enabling nanoparticles to encapsulate vaccines or metabolic-epigenetic modulators and facilitate precise and efficient delivery to targeted sites.

As a complex chronic metabolic disorder, diabetes involves not only hyperglycemia but also dysregulated lipid and protein metabolism. Whether these metabolic abnormalities synergistically activate trained immunity in conjunction with hyperglycemia requires further elucidation. Moreover, quantitative relationships between hyperglycemic stimuli and trained immunity responses—including dose-response relationships between stimulus intensity, duration, and response persistence—warrant further investigation.

The trained immunity research field has expanded from traditional immune cells to non-immune cells, such as endothelial cells, thereby broadening the scope of inquiry. This raises questions about the fundamental nature of trained immunity and the differences in molecular mechanisms between immune and non-immune cells. Addressing these scientific questions will not only advance our fundamental understanding of trained immunity but also identify more precise therapeutic targets and intervention strategies for diabetes management, potentially transforming clinical approaches to preventing and treating diabetes complications.
